# Multi-Modal Sensing for Propulsion Estimation in People Post-Stroke Across Speeds

**DOI:** 10.1109/TNSRE.2025.3577961

**Published:** 2025

**Authors:** Krithika Swaminathan, Dabin K. Choe, Daekyum Kim, Flore Barde, Teresa C. Baker, Nicholas C. Wendel, Andrew Chin, Gregoire Bergamo, Christopher J. Siviy, Christina Lee, Louis N. Awad, Terry D. Ellis, Conor J. Walsh

**Affiliations:** John A. Paulson School of Engineering and Applied Sciences, Harvard University, Cambridge, MA 02138 USA; John A. Paulson School of Engineering and Applied Sciences, Harvard University, Cambridge, MA 02138 USA; John A. Paulson School of Engineering and Applied Sciences, Harvard University, Cambridge, MA 02138 USA; School of Mechanical Engineering and the School of Smart Mobility, Korea University, Seoul 02841, South Korea.; John A. Paulson School of Engineering and Applied Sciences, Harvard University, Cambridge, MA 02138 USA; Department of Physical Therapy, Boston University, Boston, MA 02215 USA.; Department of Physical Therapy, Boston University, Boston, MA 02215 USA.; John A. Paulson School of Engineering and Applied Sciences, Harvard University, Cambridge, MA 02138 USA; John A. Paulson School of Engineering and Applied Sciences, Harvard University, Cambridge, MA 02138 USA; John A. Paulson School of Engineering and Applied Sciences, Harvard University, Cambridge, MA 02138 USA; John A. Paulson School of Engineering and Applied Sciences, Harvard University, Cambridge, MA 02138 USA; Department of Physical Therapy, Boston University, Boston, MA 02215 USA.; Department of Physical Therapy, Boston University, Boston, MA 02215 USA.; John A. Paulson School of Engineering and Applied Sciences, Harvard University, Cambridge, MA 02138 USA

**Keywords:** Estimation, gait biomechanics, machine learning, wearable sensing

## Abstract

Gait rehabilitation is critical for regaining locomotor independence after neuromotor injuries like stroke. Rehabilitation literature indicates the need for such therapy to continue beyond the clinic in order to maintain motor function and support recovery. However, implementing community-based rehabilitation requires the ability to monitor gait in the real-world with clinically relevant accuracies. Despite advances in machine learning, achieving this performance with single sensing modalities has been challenging using wearable sensors like inertial measurement units (IMUs) and pressure insoles. Here, we investigate the benefits of multi-modal sensing by integrating IMU and insole data to develop individualized machine learning models in people post-stroke that estimate propulsion, a key biomechanical variable. We show that in the lab, IMU + Insole models improve performance relative to IMU only and Insole only models, with an average root-mean-squared-error (RMSE) of 0.80 %bodyweight (%BW) across the stance phase. We obtain RMSEs of 0.71 %BW for peak paretic propulsion and 0.19 %BW s for paretic propulsion impulse, which are within corresponding clinical thresholds. We then explore the application of this algorithm to track propulsion changes in the real-world for two participants during variable-speed walking and two participants during active gait interventions, either functional electrical stimulation or exosuit-applied resistance. For these participants, we observe similar changes in measured propulsion in the lab and estimated propulsion out of the lab across speeds and interventions. Overall, this work aims to address the challenges in applying machine learning methods for individuals post-stroke and presents an investigation into the feasibility of developing estimation methods for real-world propulsion estimation during gait rehabilitation.

## I. Introduction

LOCOMOTION is a critical activity of daily living and enables independence. Unfortunately, neuromotor and traumatic injuries often impair walking ability, leading to reduced physical activity, cardiovascular health, and quality of life [1]. Stroke is a neuromotor injury and a leading cause of locomotor disability, with approximately 795,000 incidents in the United States each year [2]. The field of gait biomechanics has developed numerous methods to quantify gait kinematics and kinetics, and then mathematically characterize an individual’s movement pattern [3]. As post-stroke gait is highly variable, such quantification is particularly important to gain insight into the biomechanical contributors of specific impairments and to enable more individualized and targeted rehabilitation. A common deficit found in post-stroke gait is reduced propulsion generation in the paretic, or more impaired, leg [4]. Paretic propulsion, the anteriorly-directed component of the anterior-posterior ground reaction force (AP GRF), is correlated with high-level rehabilitation outcomes such as walking speed and clinically-determined impairment thresholds [5], [6]. In recent years, assessments based on propulsion [7] have also been frequently used to evaluate post-stroke gait rehabilitation interventions, such as robotic training or functional electrical stimulation (FES) paradigms [8], [9], [10], [11], [12]. However, measuring propulsion currently relies on force plates embedded in the floor or instrumented treadmills found in well-equipped laboratories, which limits the ability to monitor gait recovery and evaluate the efficacy of rehabilitative interventions in less controlled real-world environments. With the well-documented importance of context-relevant and continuous rehabilitation [13], there is a growing interest in enabling gait training in the community. Thus, there is an imminent need for AP GRF estimation methods that are practical for use beyond the lab, particularly for people post-stroke.

Wearable sensors are low-profile and lightweight research tools that can capture important components of gait biomechanics in such unconstrained real-world environments. In literature, two commonly used wearable sensors for estimating AP GRF during gait are inertial measurement units (IMUs) and pressure insoles [14], [15], [16]. IMUs provide three-dimensional segmental kinematic information while insoles provide unidirectional kinetic information representing the normal component of loading. Both sensors have primarily been individually integrated into algorithms for healthy populations during short walking bouts [17], [18], [19], [20] and more recently for people post-stroke during overground walking [10], [21], [22]. IMU-based AP GRF estimation methods are popular given their relatively lower hardware costs, and have achieved estimation errors as low as 3.4 %body-weight (%BW) in unimpaired individuals [19] and 2.6 %BW in people post-stroke [21]. However, these approaches assume that kinematics are robustly related to kinetics, which may be less valid for people post-stroke [23], [24]. On the other hand, high-resolution pressure insoles may be more effective than IMUs for estimating AP GRF as they provide a direct, local measure of loading at the foot. Prior work has shown that in unimpaired individuals, insoles have been able to achieve estimation errors below 3 %BW for AP GRF [25], [26]. However, insole use for propulsion estimation in people post-stroke remains limited, with the best reported performance resulting in errors of 6.4 %BW [27]. This lack of use may be because insoles only measure vertical loading, whereas clinically relevant propulsion metrics rely on shear loads along the anterior-posterior axis that may require extra information or more complex methods to accurately estimate [22]. Given that IMUs and insoles provide fundamentally different information, we hypothesized that leveraging both kinematic and kinetic data may improve estimation performance, as previous work has shown benefits of incorporating data from diverse sources [28]. Combining IMU and insole data in unimpaired individuals has shown promising results in improving estimation accuracy of other biomechanical metrics, such as tibial loading metrics during running [29]. However, such sensor fusion for estimating biomechanical metrics in people post-stroke, such as propulsion, remains unexplored.

Estimation approaches for AP GRF, both with IMUs and insoles, can be broadly categorized into physics model-based methods and data-driven machine learning methods [16]. In people post-stroke, there is high variability within and across individuals, and biomechanical relationships between sensor data and AP GRF may deviate from normative gait patterns [23], [30]. Thus, recent work has focused on developing individualized data-driven models instead of physics-based models to directly map sensor data to ground truth without physical constraints [21], [22]. Data-driven models can range in complexity from linear regression, which is lightweight and suitable for sensors that are correlated with the target metric, to non-linear deep neural networks, which can capture abstract relationships between sensor data and the target metric but require more intensive computation. Indeed, our prior work using pressure insoles showed the benefit of neural networks to estimate planar loads, while linear regression was sufficient to accurately capture vertical loads [22]. Although data-driven approaches are promising, the limited walking capacity of people post-stroke poses a significant challenge for building sufficiently extensive datasets for both individualized and generalized models [31]. As a result, data-driven methods, including individual-specific models, are often less generalizable to unseen conditions and datasets [32], [33].

In this work, we posit that incorporating multi-modal sensing can improve the performance of individualized machine learning-based models across a range of walking conditions relevant for real-world use by capturing individual-specific relationships between kinematics and kinetics. Specifically, post-stroke gait is affected by walking speed and environment [34], and by rehabilitation interventions [35]. Given their similarity to community-based walking, overground walking datasets are widely preferred for model development, training, and testing. However, due to the challenging nature of overground biomechanics collection, these datasets are also limited in size, both in number of strides and in the range of walking speeds represented [21], [27]. This lack of breadth in the data can subsequently lead to the diminished ability of models trained with these smaller overground datasets to reliably estimate metrics in real-world scenarios. Conversely, instrumented treadmills simplify data acquisition but are often set to a constant speed, resulting in datasets with more strides at the cost of reduced speed variability. Thus, there is potential for data collected at various speeds and with various rehabilitation interventions on a treadmill to augment input datasets and train machine learning models for overground and out-of-lab environments.

The objective of this work was to investigate the use of a multi-modal machine learning method to estimate AP GRF and propulsion metrics in people post-stroke for future use in assessments of rehabilitation interventions. We first evaluate the efficacy of combining IMU and insole data to estimate propulsion during walking at variable speeds, compared to Insole only and IMU only models, in both unimpaired individuals and people post-stroke (Section III. A). We then investigate the performance of the estimator with reduced training datasets across different input modalities (Section III. B). Recent work has further suggested the promise of transfer learning methods to augment individual-specific training data with group-level data [10] to reduce the burden of data generation for any one individual. Thus, we also investigate the potential to augment smaller clinical datasets with more readily available unimpaired data to improve performance of individualized models through transfer learning [36] (Section III. B). Finally, we conduct a series of proof-of-concept application-centric demonstrations to evaluate the performance of these individualized models under mostly unseen walking conditions such as overground and out-of-lab walking (Section III. C), and during gait training with wearable systems, such as FES and a soft resistive ankle exosuit (Section III. D).

## II. Methods

### A. Participants

We collected data from a cohort of six individuals post-stroke in the chronic phase of recovery (>6 months post-stroke, 1F, 5M; age: 54 ± 9 yrs (mean ± std); height: 176 ± 8 cm; weight: 98 ± 17 kg) and seven unimpaired adults (4F, 3M; age: 28 ± 4 yrs; height: 176 ± 11 cm; weight: 72 ± 21 kg) (see Table I for details). In our clinical cohort, four participants were left paretic and two participants wore a rigid ankle-foot-orthosis. One participant completed two visits, leading to a total of seven clinical datasets (*D*_1*C*_,…*D*_7*C*_). We aimed to represent a broad range of impairment levels in the group, and thus, the comfortable treadmill walking speeds of the participants ranged from 0.5 to 1.0 m/s (0.71 ± 0.22 m/s). All unimpaired participants reported no previous history of musculoskeletal injury or disease. The study was approved by the Harvard Longwood Medical Area Institutional Review Board and all individuals provided medical clearance and written informed consent.

### B. Experimental Protocol: Main Study

**Fig. 1.**
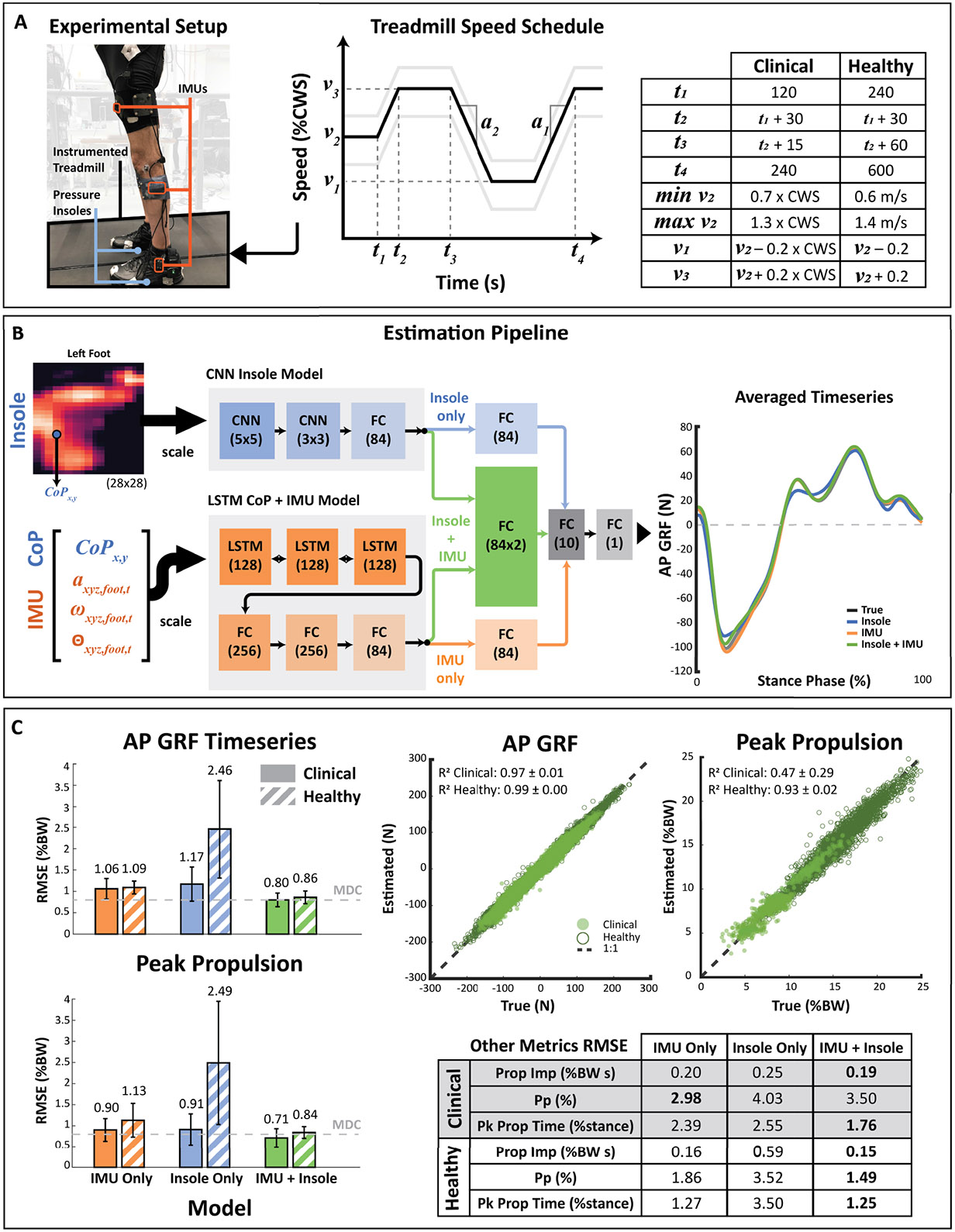
**A.** Experimental protocol with treadmill speed schedule. **B.** (Left) Multi-modal estimation pipeline. (Right) Sample averaged time series data across the stance phase for all strides from the participant with the median average RMSE in our clinical cohort. Averages are plotted for the estimates from the three models along with the ground truth values. **C.** (Left) Performance of individual-specific models across the stance phase with different sensor combinations. Bar plots represent mean RMSE and the error bars represent standard deviations. (Right, Top) Correlation plots for AP GRF time series data and peak propulsion for both clinical and healthy participants. For AP GRF time series data, we sample every 100th point from the healthy test sets and every 50th point from the clinical test sets to improve the interpretability of visualization. R^2^ values listed in the legend represent the mean ± std across all datasets. (Right, Bottom) Average RMSE performance across individual-specific models for estimating point metrics with different sensor combinations. Bolded entries represent the best combination for the corresponding metric and cohort. All data represent performance on the paretic side in people post-stroke and on the left side for the unimpaired cohort.

Participants walked on an instrumented treadmill (Bertec, Columbus, OH, USA; 2000 Hz) at a range of speeds relative to a comfortable treadmill walking speed (CWS). The CWS for clinical participants was determined at the start of the session by slowly ramping up the treadmill speed until the participant reported the speed to be too fast and then returning to the previous comfortable speed. For each walking bout, the treadmill was programmed using a custom Simulink (Mathworks, Natick, MA, USA) script to command pre-set speed profiles to introduce speed variability both within and across walking bouts. The commanded profiles were held at a constant speed for an initial period and then ramped up and down through a predetermined range of speeds for the remainder of the walking period (see Fig. 1A for treadmill speed profiles). Clinical participants walked for three to six 4-minute bouts (*t*_4_ = 240 s), depending on the individual’s impairment and endurance levels (Table I), in which the first 2 minutes were at a constant speed (*t*_1_ = 120 s). Unimpaired participants walked for six 10-minute bouts (*t*_4_ = 600 s), in which the first 3 minutes were kept constant (*t*_1_ = 180 s). For the clinical cohort, the speed of each bout was centered around a different percentage of the CWS (*v*_2_ = 70–130 %CWS) [in increments of 10% CWS], and the order of bouts was randomized. For the unimpaired cohort, the initial speed (*v*_2_) of each trial ranged from 0.6–1.4 m/s [in 0.2 m/s increments] to capture the range of speeds observed in our clinical cohort and were also applied in a randomized order. The minimum and maximum speeds were defined as a function of the initial speed such that *v*_1_ = *v*_2_ – 20 %CWS and *v*_3_ = *v*_2_ + 20 %CWS for clinical participants, and *v*_1_ = *v*_2_ – 0.2 m/s and *v*_3_ = *v*_2_ + 0.2 m/s for healthy participants. These minimum and maximum speeds were held for 15 s at a time by the clinical cohort and 60 s at a time by the healthy cohort. A seated rest was provided between bouts if needed. Half of the participants ramped up in speed first (*a*_1_ > 0), while the remaining participants ramped down in speed first (*a*_1_ < 0) to account for any effects of acceleration versus deceleration. Specifically, acceleration of the ramp sections was commanded such that the speed changed by *v*_2_ – *v*_1_ in 30 seconds.

We measured bilateral lower limb kinematic data from IMUs (MTi-3, XSens, Enschede, Netherlands) placed on the feet, shank, and thigh segments at 100 Hz via Bluetooth. Specifically, we obtained segmental orientation angles, 3-dimensional angular velocities, and 3-dimensional local acceleration measurements from each IMU. We also used an optical motion capture system to track kinematics of both legs, which were later used for syncing data sources (Qualisys, Gothenburg, Sweden; 200 Hz). Participants wore commercial pressure insoles on both feet (XSensor, Calgary, AB, Canada; 50 Hz), which streamed pressure data from 233 individual sensels (sensing cells) per foot and the center of pressure (CoP) coordinates. We collected AP GRF data at 2000 Hz from force plates embedded in the treadmill throughout the walking bouts to serve as ground truth measurements.

### C. Data Pre-Processing

The insole data were first normalized by identifying a time point when the foot was off the ground and zeroing all sensels. Then, both the insole and IMU data were low-pass filtered using a zero-phase, 2nd order Butterworth filter with a 10Hz cutoff frequency to remove noise artifacts. The same filter was also applied to the force plate data. We additionally applied a spatial Gaussian filter to the insole data using a standard deviation *σ* of 0.5 to reduce sensitivity to temporary pressure points. The standard deviation was selected after a grid search with *σ* ∈ {0, 0.1, 0.25, 0.5, 1, 2} and to be consistent with prior literature [37].

After filtering, we time-aligned all data sources. We first identified heel strike events using force plate and insole pressure data independently. Then, stride-by-stride insole data were interpolated to match timestamps obtained from the force plate data. The IMU data were synchronized with the motion capture data, and re-interpolated to match the force plate timestamps. Only signals that were present in all participants’ data were used as input for the models; thus, we discarded thigh acceleration data. We further discarded yaw orientation angles from all limb segments given the known challenges of drift in this signal over time [38]. A total of 21 signals were used from the IMUs: 3D local acceleration from the foot and shank; 3D angular acceleration from the foot, shank, and thigh; and pitch and roll Euler angles from the foot, shank, and thigh. For the foot and shank segments, the IMUs were placed such that the pitch angle corresponded to the sagittal plane kinematics, while for the thigh segment, the IMUs were placed such that the roll angle aligned with the sagittal plane. Finally, all data were resampled to 100 Hz.

We conducted two additional pre-processing steps prior to using this data as input to the machine learning models. First, we subtracted the average signal captured during the swing phase of the first stride in each trial from the insole data, which we assumed should theoretically be zero in the absence of noise. We then resized the insole sensel array into a 28 × 28 square map using pixel area interpolation in OpenCV [39] to enhance compatibility with machine learning methods for image processing, i.e., convolutional neural networks [40].

Trials in which any of the data were missing due to technical complications (e.g., dropped Bluetooth packets) were excluded from further analysis. The number of trials used from each dataset is provided in Table I.

### D. Model Development

#### 1) Model Architecture:

To fuse the input data while also learning sensor-specific patterns, we used an architecture with separate networks for each input mode that are merged at a later stage (Fig. 1B). In our prior work, we found that a convolutional neural network (CNN) is well-suited for pressure insole data given its image-like structure, and thus, we chose to use the same network architecture given its demonstrated efficacy [22]. Briefly, the network used for the insole data in this work, and described in Bergamo et al. [22], contained two convolutional layers with kernel sizes of 5 and 3, with average pooling layers between the convolutional layers. The convolutional layers were followed by a fully-connected (FC) layer with 84 neurons. The IMU and CoP data have a time series structure, with prior data informing future states. Long Short-Term Memory networks (LSTMs) are a form of recurrent neural networks that are widely used to predict information using time series data [41], [42]. Thus, we used an LSTM architecture to learn the underlying structure of the IMU and CoP time series data, using sequences of 5 timesteps, corresponding to approximately 50 ms periods. The sequence length was determined by considering the tradeoff between model performance on a validation set (see: II-D.2) and the increase in model complexity. The LSTM comprised three bidirectional LSTM layers with 128 hidden features each, followed by two FC layers with 256 neurons each and one FC layer with 84 neurons. The outputs of the final FC layers from the insole and IMU networks were then merged as input to an additional FC layer with 168 neurons. Last, we added an FC layer with 10 neurons to map to ground truth AP GRF data at the corresponding frame. We used rectified linear unit (ReLU) activation functions for the output of each layer prior to the final FC layer to capture nonlinearity in the data. In the Insole only and IMU only models, the last sensor-specific output was fed into an FC layer with 84 neurons instead of 168 neurons and all other layers were unmodified. Of note, CoP data was only used in the Insole only and IMU + Insole models. Fig. 1B depicts the estimation pipeline for the “Insole only,” “IMU only,” and “IMU + Insole” configurations.

#### 2) Train-Validation-Test Splits:

To train these individual-specific models, we separated each dataset into training, validation, and test sets by walking bout. The validation sets were used to select the final set of model weights. Specifically, the walking bouts closest to the comfortable walking speed were used as the validation and test sets, while all remaining walking bouts were used as training data. Both IMU and insole data were normalized to the range observed in the training dataset using min-max normalization. IMU data were normalized by sensor and signal (e.g., 3D acceleration for the foot IMU were normalized together). Data corresponding to the swing phase were removed to prevent overfitting to a region where AP GRF is consistently zero. Similar to our prior work, we used the Adam optimizer with a learning rate of 5e-4 and a weight decay of 1e-5, and the mean squared error loss to train each model over 500 epochs [22]. All neural network code was written using Pytorch 2.1.2 [43].

For all clinical datasets, we also explored the training data requirements for accurate estimation by varying the datasets used to train the models. We investigated the total time of walking required to train the models for clinical datasets, independent of the walking speed. For each clinical dataset, we combined data from all walking bouts and varied the ratio of data used in the training set. Specifically, we used the first 15%, 30%, 45% and 60% of each walking bout as the training set, the next 10% for validation, and the remainder as the test set. For each trial, 15% is approximately 30 seconds of walking. We note that in this approach, the constant regions of all walking bouts are represented in the training set, with the 60% condition also including some of the varying speed periods. Then, we investigated using the same ratios, but by using the last section of the walking trials as training data. This split then increased the range of speeds in the training datasets without increasing its size.

#### 3) Transfer Learning:

As another approach to tackle the challenges of small datasets with clinical populations, we investigated the potential of leveraging unimpaired data to supplement clinical estimates. We implemented transfer learning by using all healthy datasets for pre-training and then fine-tuning to create the final individual-specific models. Specifically, all training data from healthy individuals were combined and used to pre-train the same multi-modal estimation model architecture for 1000 epochs (Fig. S1). The last weights from pre-training were used to initialize the individualized model and represent the best performing weights on the healthy training data. Then, the model was fine-tuned on the paretic side data from each clinical dataset for 500 epochs. This fine-tuned model was evaluated on the same intermediate walking bout as in the main investigation. We evaluated the relative performance of models with and without fine-tuning using both, 100% and the last 50%, of the training and validation data, while the test dataset remained unchanged.

### E. Evaluation Metrics

We evaluated model performance across all sensor input combinations using root-mean-squared error (RMSE) scaled to the participant’s bodyweight, and the coefficient of determination (R^2^) between the estimated and true AP GRF values during stance. We additionally computed normalized RMSE (NRMSE) as the RMSE scaled to the range of the test data during the stance phase. To test the hypothesis that multi-modal models would outperform single-mode models, we conducted pairwise *t*-tests between the IMU + Insole and IMU only models, and between the IMU + Insole and Insole only models. The *t*-tests were performed separately to evaluate the performance of these models across all clinical and healthy datasets. Model errors from both legs were pooled for both cohorts. To determine the practicality of the approach, we also assessed the model’s performance in estimating key point metrics of paretic propulsion that have been linked to impairment levels in people post-stroke: peak propulsion magnitude, propulsion impulse, and paretic propulsion symmetry [44]. Peak propulsion was defined as the maximum AP GRF. Propulsion impulse calculations were performed by time-integrating the positive region of AP GRF during stance. Propulsion symmetry was computed as the ratio between the paretic propulsion impulse and the total propulsion impulse from both limbs, with 50% representing perfect symmetry [4]. We compared RMSE values to the minimal detectable change (MDC), which represents the expected variability in a population for a given activity, during treadmill walking for people post-stroke [45]. Finally, we also evaluated errors in the timing of peak propulsion as a percentage of the stance phase. We focused on the left leg for all healthy participants, assuming bilateral symmetry, and the paretic leg in people post-stroke given our target application.

### F. Experimental Protocols: Proof-of-Concept Application-Centric Demonstrations

After evaluating the estimation performance of the trained models, we conducted a series of proof-of-concept exploratory experiments to assess the feasibility of deployment in real-world environments. For all experiments, the same measurements were collected and the same post-processing pipelines were used as in the main study unless otherwise stated.

Given that the model was developed using treadmill data, we were interested in evaluating its ability to predict data during walking in unconstrained environments. Three people post-stroke (> 6 months post-stroke, 3M; age: 55 ± 14 yrs; height: 181 ± 4 cm; weight: 81 ± 14 kg) were invited for a separate single-session study. The three individuals (S3, S6, and S14) presented with varying baseline comfortable walking speeds, ranging from 0.35 m/s to 0.95 m/s, to represent a wide array of impairment levels. Participants completed three treadmill walking bouts, three overground walking bouts, and three out-of-lab walking bouts, one each at their self-selected slow, comfortable, and fast walking speeds. The treadmill bouts followed the same scheduling as in the main study. The model was trained on all treadmill bouts and the slow overground walking bout. The fast overground walking bout was used for validation, and the last overground walk, at their comfortable speed, was used for testing. We then estimated changes in paretic propulsion as participants walked along an out-of-lab straightaway and were asked to vary their speed at 10 m increments. One participant (S6) was only able to complete two treadmill trials and one overground trial due to fatigue. For this individual, we trained and validated the model using the treadmill data and tested on the overground data.

Then, we aimed to assess the utility of this approach to track propulsion during active gait training. We brought in three participants post-stroke for single-session data collections (> 6 months post-stroke, 1F, 2M; age: 64 ± 4 yrs; height: 173±6 cm; weight: 87 ± 32 kg). Participants (S5, S14, and S15) completed two treadmill walking bouts, one overground walking bout, and one out-of-lab walking bout, all at a constant self-selected comfortable speed. Each bout was four minutes long and comprised one minute of unperturbed baseline walking, two minutes of active intervention, and one minute of unperturbed post-intervention walking performed in sequence. Treadmill speeds were kept constant to prevent instability from concurrent changes in speed and intervention state for the participant, as well as to reduce confounding factors in propulsion changes during analysis. Two individuals received resistive exosuit training, which has been shown to influence propulsion within two minutes of training [12]. One individual received FES, which has also shown propulsion benefits in this population [9], [46]. Parameters of the exosuit-applied resistance and FES were determined prior to the start of the walking trials using previously established techniques [12], [47]. The model was trained and validated on the treadmill bouts and tested with the overground walking bout. We used the trained model to track paretic propulsion during the first baseline period (one minute) and the active intervention period (two minutes).

## III. Results

### A. Effects of Multi-Modal Sensor Input Across Participants

We first investigated the performance of each sensor input combination. We find that in both healthy and clinical cohorts, the average test RMSE across the stance phase is usually improved through multi-modal sensor inputs (Fig. 1C, Table S1). In our healthy cohort, the multi-modal model architecture performed best for six out of seven datasets, typically followed by the IMU only and Insole only models. Across datasets, the average RMSE with the IMU + Insole model was 0.86 ± 0.15 %BW, versus 1.09 ± 0.15 %BW with the IMU only model and 2.46 ± 1.15 %BW with the Insole only model. The RMSE of the IMU + Insole model was significantly lower than those of the Insole only (*p* < 0.001) and IMU only (*p* = 0.009) models across both legs. Similarly, the NRMSE is minimized at 1.98 ± 0.27% and R^2^ is maximized at 0.99 ± 0.00 with the IMU + Insole model. We find that in people post-stroke, the IMU + Insole model performed best for six out of seven datasets, while performance between IMU only and Insole only models varied across datasets. Specifically, the IMU + Insole model resulted in an average RMSE of 0.80 ± 0.16 %BW, versus 1.06 ± 0.24 %BW and 1.17 ± 0.40 %BW with IMU only or Insole only models, respectively. The RMSE of the IMU + Insole model was significantly lower than those of the Insole only models (*p* = 0.034) and trended towards significantly lower than those of the IMU only models (*p* = 0.055). On average, correlations between the estimated and true AP GRF were also strongest with the IMU + Insole models, followed by the IMU only and Insole only models. These results are also consistent with the contralateral non-paretic side in the clinical cohort and the right side in the healthy cohort (Table S2).

Moreover, this improvement in estimator performance across the time series translated to increased accuracy in capturing key clinical point metrics (Fig. 1C), with an average RMSE of 0.71 ± 0.22 %BW in peak propulsion and 0.19 ± 0.07 %BW s in propulsion impulse during stance, both of which are within the MDC thresholds for treadmill walking of 0.80 %BW and 0.24 %BW s, respectively [45]. Propulsion symmetry was also estimated with an RMSE of 3.50 ± 2.44% with the IMU + Insole models, which is below the corresponding MDC of 3.92%. We further find that these models capture the timing of peak propulsion to within 2% of the stance phase. For the IMU + Insole models, estimated versus true point metrics are plotted for each dataset in Fig. S2, and errors for each clinical dataset are provided in Table S3.

### B. Effects of Multi-Modal Sensor Input Across Training Datasets

**Fig. 2.**
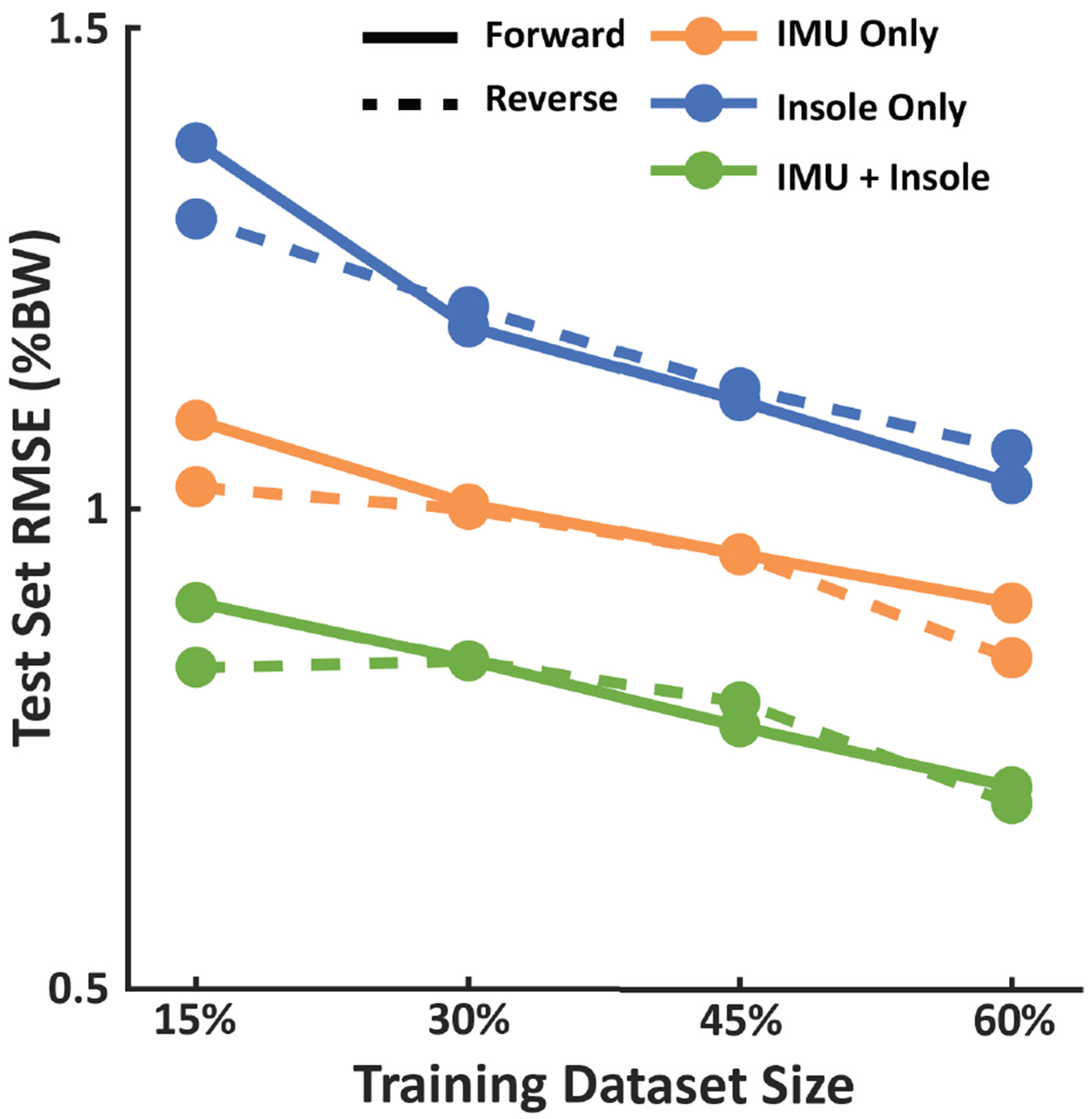
Average effect of training dataset size and speed variability across our clinical datasets ranging from 15% to 60% of all the walking trials. Solid lines indicate using the start of each walking bout (“Forward” split), which primarily contains constant speed walking, while dashed lines indicate using the end of each walking bout for training (“Reverse”split), which contains both varying and constant speed walking.

We observed that the benefit of using multi-modal input was also preserved when using smaller proportions of each walking trial for training (Fig. 2). On average, with the first 15% (~30 s) of each trial, RMSE was 0.90 %BW with IMU + Insole compared to 1.09 %BW and 1.38 %BW with IMU only and Insole only, respectively. Similarly, with the first 60% of each trial, RMSE was 0.71 %BW with IMU + Insole compared to 0.90 %BW and 1.03 %BW with IMU only and Insole only, respectively. We also found that for the 15% training datasets, introducing more speed variability in the training data improved performance compared to datasets of equal size with less variability. Specifically, by using the speed ramp sections at the end of the trial (“Reverse” in Fig. 2) rather than the constant speed sections at the start of the trial (“Forward” in Fig. 2), estimation errors were reduced. For example, with the IMU + Insole model, using the last 15% of the trials resulted in an RMSE of 0.83 %BW compared to 0.90 %BW with the first 15%, and this trend is present in the IMU only and Insole only models as well.

We also found that pre-training the models on our healthy dataset and then fine-tuning on half the dataset for each individual reduced average estimator error across the AP GRF time series data from 0.93 %BW to 0.86 %BW (Table S4). We observed reductions in error of more than 0.1 %BW for two out of seven datasets, changes within 0.1 %BW for four datasets, and an increase of more than 0.1 %BW for one dataset. Changes in peak propulsion estimation accuracy aligned with the time series estimation performance. Conversely, applying transfer learning to the full dataset led to negligible changes relative to the base individualized model (Table S5).

### C. Demonstration of Propulsion Estimation Across Varied Environments

**Fig. 3.**
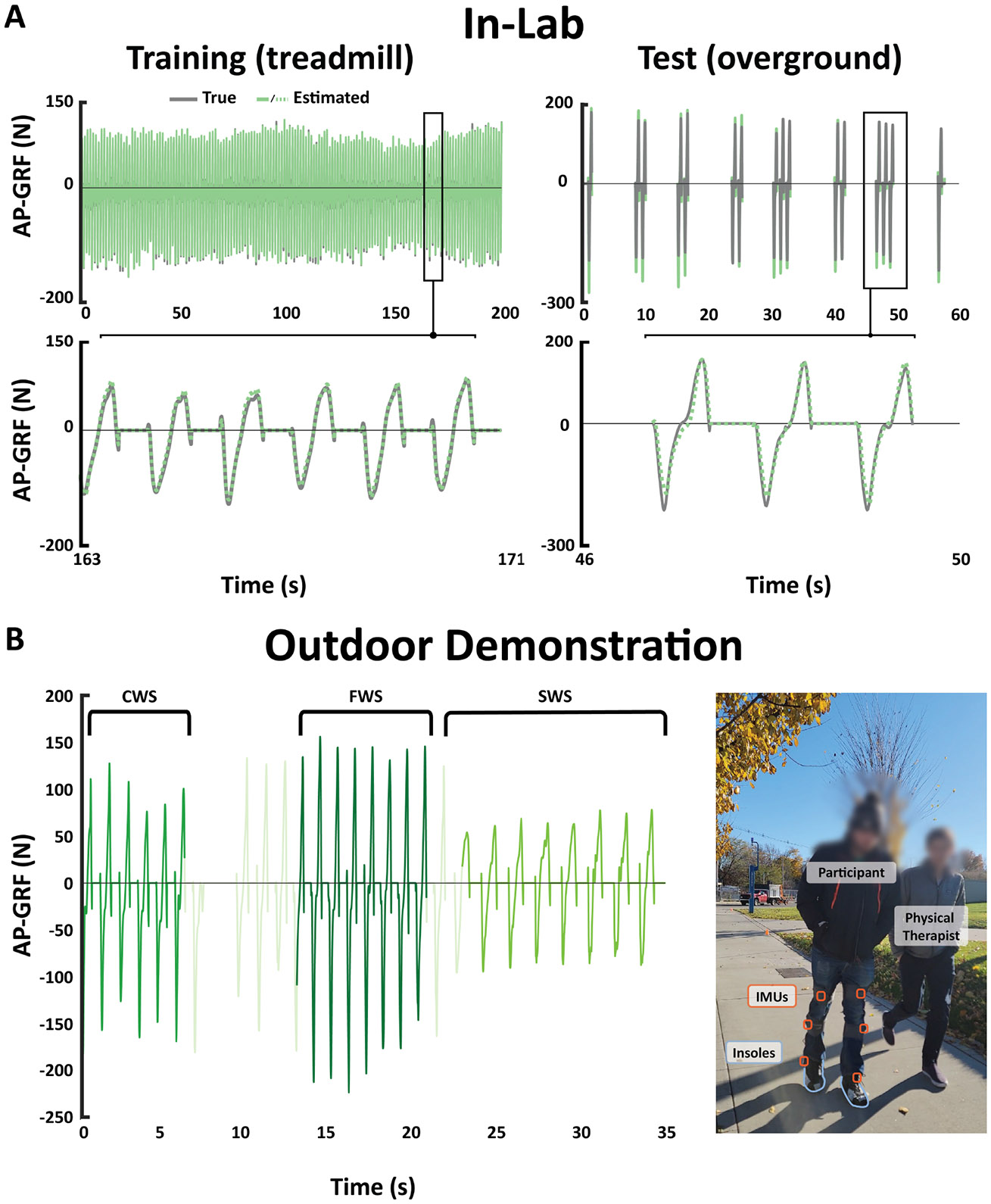
Demonstration of estimator performance across different environments for a sample participant. Models were trained using three trials of treadmill walking (slow: SWS, comfortable: CWS, and fast: FWS) and one overground trial, and validated and tested on overground trials. We then estimated AP GRF in out-of-lab environments as individuals varied their speed every 10 m. For this participant, we observe that the estimator also captures a halt in walking around 8 s into the outdoor trial due to a toe cramp.

In our exploration of predicting propulsion during walking in unconstrained environments, we found that propulsion estimation accuracy was reduced during overground walking after training the models primarily on treadmill walking. However, using multi-modal estimation still resulted in slightly improved transfer to overground walking, with an average RMSE of 1.95 %BW, compared to 2.03 %BW with IMU only and 2.68 %BW with Insole only (Table II). As there is no ground truth outside of the lab, we compared out-of-lab estimates of peak propulsion across participants’ self-selected slow, comfortable, and fast speeds with true peak propulsion obtained from force plates during overground walking in the lab, also at participants’ self-selected speeds. Changes in propulsion estimates during walking in real-world environments mirrored changes in ground truth propulsion observed within the lab (Table II, Fig. 3). For example, in the lab, S3 increased speed by 0.72 m/s (slow to fast walking) with a corresponding increase in peak paretic propulsion of 10.57 %BW. Similarly, along an outdoor sidewalk, this participant increased walking speed by approximately 0.54 m/s with a corresponding estimated increase in peak paretic propulsion of 7.76 %BW. For S14, we found that an increase in speed from 0.76 to 0.97 m/s (slow to medium) was associated with an increase in peak propulsion of 1.57 %BW. In a hallway outside of the lab, we estimated that an increase in speed from 0.70 to 0.93 m/s (medium to fast) was associated with an average increase in peak propulsion of 1.52 %BW.

### D. Tracking Propulsion During Active Gait Training

Then, we aimed to assess the utility of this approach to track propulsion during active gait training with either a resistive exosuit or an FES neuroprosthesis. Consistent with our prior tests, we found that AP GRF estimation accuracy during gait training was maximized by the IMU + Insole models (Table III). This improvement was consistent both with and without active external intervention via a soft wearable exosuit (S5 and S14) or FES (S15).

We again compared ground truth changes in peak propulsion in the lab with out-of-lab estimates. We found that our estimates of peak propulsion changes from the baseline to the active periods were similar between in-lab overground walking and out-of-lab walking for two participants, S14 and S15 (Table S6). Specifically, for S14, peak propulsion during active exosuit resistance was 1.13 %BW larger than during the baseline period in the lab. During out-of-lab level-ground walking, estimated peak propulsion during active exosuit resistance was 1.19 %BW larger than during the baseline period. Similarly, for S15, we found a decrease of 0.15 %BW in peak propulsion during active FES during in-lab overground walking compared with an estimated decrease of 0.14 %BW during out-of-lab walking. S5 did not have any usable data from the force plates during the in-lab overground section for comparison.

**Fig. 4.**
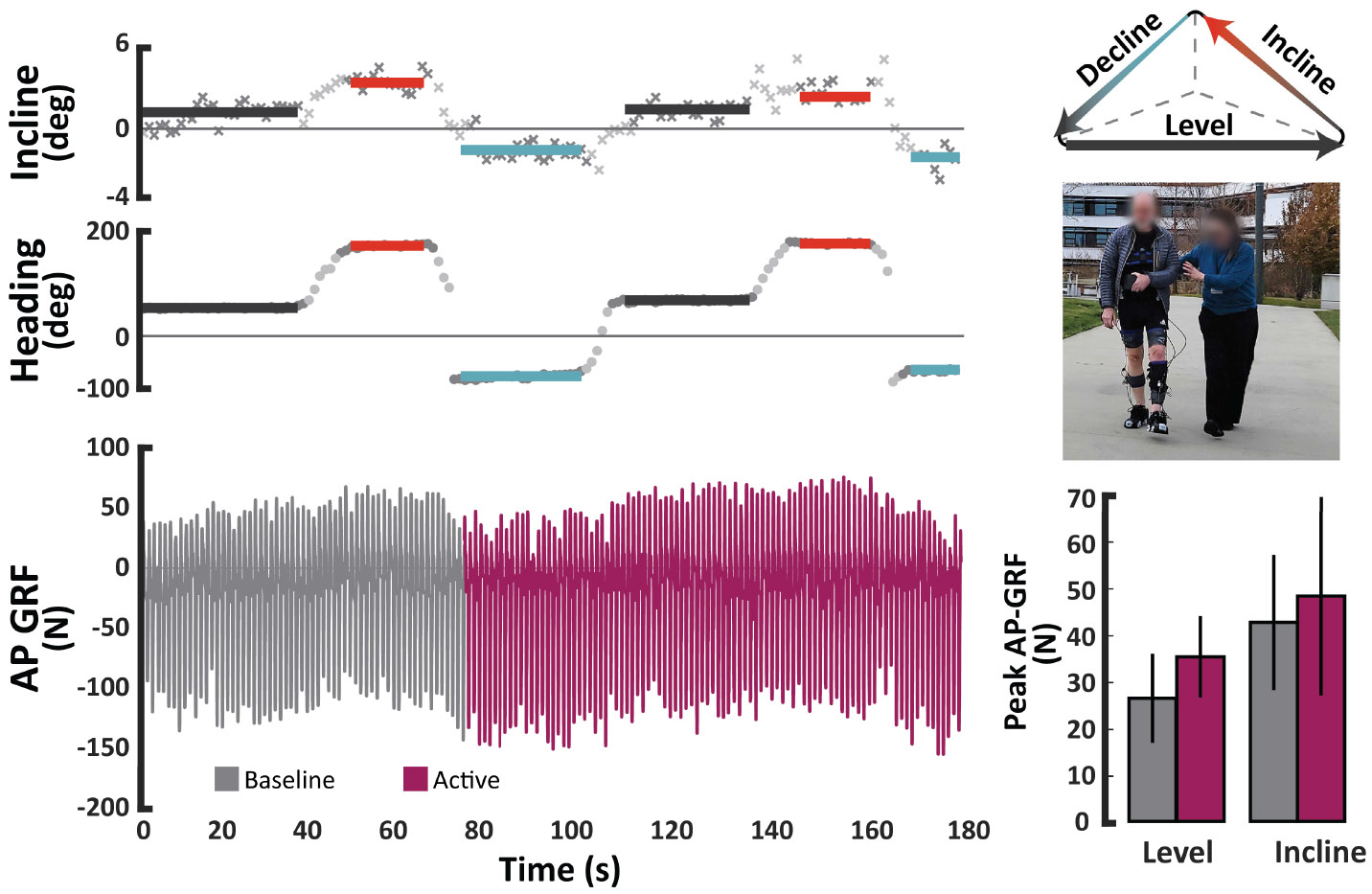
Tracking paretic propulsion with and without active exosuit-applied resistance on an outdoor triangular path. The path was not perfectly flat, with each side having a slight incline or decline. We observe that the estimated propulsion reflects changes in accordance with the incline of the path as well as in response to the active section of training.

We also observed that the model was sensitive to environmental changes during outdoor walking. For example, S14 walked around an inclined triangular pathway while the soft exosuit alternated between being transparent and applying active resistance. We observed changes in propulsion estimates that corresponded both with the slope of the walkway and with the exosuit state (Fig. 4). Further investigation is required to decouple the relative effects of the environment from the intervention.

## IV. Discussion

In this work, we demonstrate clinically relevant propulsion estimation for people post-stroke by leveraging kinematic and kinetic information from IMUs and pressure insoles (Section III. A). We show that multi-modal estimation improves generalizability of the learned models to new walking speeds and environments across individuals (Section III. C). Moreover, by using a multi-modal model architecture, we observe improved performance with small datasets, which suggests its increased utility for populations with higher fatigability, who may not be able to walk to generate larger training datasets (Section III. B). We show that for a subset of our cohort, we can further mitigate the challenges of smaller datasets by applying transfer learning techniques between healthy and post-stroke datasets. Finally, we demonstrate the potential for this multi-modal estimation approach to enable monitoring of paretic propulsion in unconstrained environments and during active gait rehabilitation (Section III. D).

### A. Multi-Modal Models Enable Accurate Estimation in People Post-Stroke

A unique challenge to developing estimation methods for individuals post-stroke is the relatively small magnitudes of propulsion generated on the paretic limb and the correspondingly small changes that are clinically relevant [44]. Consequently, there are limited prior benchmarks for post-stroke propulsion estimation. To date, the best reported propulsion estimation accuracy for subject-specific models in people post-stroke is an error of 1 %BW with an IMU only model that uses a full stride of kinematics data to predict AP GRF, including the swing phase, introducing an estimation delay of one stride [10]. With the multi-modal architecture, we achieved improved performance with errors below the corresponding minimal detectable change (MDC) thresholds for peak propulsion, propulsion impulse, and propulsion symmetry [45]. The MDC represents a measure of variability within the population, and thus these results suggest that our method can capture any change in an individual that is not just from stride-to-stride variability. Moreover, by estimating sample-by-sample data, our approach allows for high accuracy in estimation with negligible latency in predictions, suggesting its potential for use in active rehabilitation applications.

### B. Multi-Modal Models Improve Estimation Across Environments in People Post-Stroke

We further showed that multi-modal estimation was best across varying speeds and environments, both of which often vary throughout the course of rehabilitation and recovery as mobility levels evolve [48], [49], [50]. Given that both loading and kinematic variables are modified as walking speed changes, this result may reflect the model’s ability to encode the various contributors to walking speed [51] and extrapolate to other unseen conditions. Similarly, we found that using both sources of information allowed for improved performance during overground walking. In our out-of-lab demonstrations, we found that the IMU + Insole model captured the same magnitude and direction of change in peak propulsion relative to different self-selected walking speeds in and out of the lab. While we do not have access to ground truth outside of the lab, this approach of validation using correspondence between changes observed in the lab across different walking conditions and those estimated outside of the lab has also been used in prior work [10], [52]. IMUs are commonly used in translation-focused estimation methods as users are often already in possession of these sensors in the form of phones and fitness trackers. Indeed, prior work has shown the ability of a single IMU at the pelvis to capture multiple kinematic and kinetic features of gait in healthy individuals [53]. However, in addition to increased stride-by-stride kinematic variability [23], people post-stroke can also experience tremors [54] that introduce additional noise into IMU data. Moreover, IMU calibration and placement sensitivity are known challenges in the field [55], [56], and the increased presence of non-sagittal plane motion, such as gait compensations [57], may further complicate the relationship between IMU placement and kinetic measurements.

Although we did not directly test over very long durations (i.e., multiple days), our findings suggest that directly capturing loading measurements in addition to IMU measurements improves AP GRF estimation, even with the slight shifts in sensor placement that may occur over the course of a few hours. While the increased dimensionality of the input data may contribute to these performance improvements, prior work has shown that combining multiple sensor types is important for accurately estimating gait biomechanics in healthy individuals [58]. Multi-modal estimation has also been shown to improve model generalizability to new contexts in other domains, such as running and health monitoring [29], [59], and thus our work aligns with these principles in the clinical gait biomechanics domain. An increasing number of studies are investigating the efficacy of community-based rehabilitation interventions with and without wearable systems in various populations [10], [60], [61], [62]. This work suggests that with a combination of wearable sensors, changes in key metrics can be reliably estimated and used to continuously evaluate efficacy of these interventions outside the lab.

### C. Multi-Modal Models Improve Propulsion Estimation With Scarce Datasets

This generalizability to new walking conditions is particularly important for people post-stroke where obtaining long periods of walking data presents a challenge but where gait often changes over time with physical therapy or muscle atrophy. While most prior literature uses overground walking datasets to better represent overground walking in the real world [63], they are limited in size. We aimed to mitigate this scarcity of data by using treadmill data to train our model and modulated speed both during and across walking bouts to introduce more variability into the dataset [64]. Acknowledging the complex relationship between dataset size and network complexity [65], we also investigated model performance after training on smaller proportions of the training datasets and found that multi-modal models consistently outperformed Insole only and IMU only models. This result may be due to multi-modal sensing supplementing the lack of data with richer, higher dimensional information. We further found that increasing variability in the training set by including more of the speed-ramp periods than the constant-speed periods reduced estimation error with the smallest training set investigated. We suggest this may be partially due to the fact that more speeds were included in the training set, but also due to the learned weights reflecting the trends between the IMU and insole data, walking speed, and AP GRF output. These results align with prior work in machine learning literature that has shown that including small amounts of out-of-distribution data in the training set improves overall model performance [66], [67]. Our smallest dataset used approximately 25% of all the data collected for a given individual for model training and validation, which corresponds to an average walking duration of about 4.5 min per individual. This duration is less than the commonly used 6-minute walk test [68] and is feasible for most ambulatory people post-stroke.

Although a generalized model would require no data from an unseen individual and increase accessibility for people post-stroke, the amount of data required remains a limitation as individuals use a variety of assistive devices and exhibit a wide range of gait patterns, and may be addressed in the future as large biomechanics datasets grow in the community [69], [70]. Instead, an individualized model that uses short walking durations to estimate kinetics in a broad range of contexts may be most clinically appropriate. For individuals for whom obtaining sufficient data to train a subject-specific model presents a challenge, we can augment the training dataset with data from other individuals and speeds as pre-training and apply transfer learning methods [36] to improve estimation accuracy. We used the healthy dataset, with approximately 7 hours of walking data, to pre-train each individual model, and observed substantial reductions of at least 0.1 %BW in estimation errors for a subset of individuals. However, these improvements were only evident when using half of the individual-specific fine-tuning dataset (~12 min instead of 24 min), suggesting that transfer learning is only helpful when there is limited data in the target domain, similar to prior uses of transfer learning for medical applications [71], [72]. Individuals for whom estimation performance improved from fine-tuning were independent of impairment level, paretic side, and dataset size, suggesting that the healthy data contained relevant information for a wide range of gait presentations, but that further investigation is required to understand which individuals benefit most from leveraging transfer learning. This initial characterization provides a benchmark for future studies to use to decide whether to collect the necessary data to pre-train a model based on their accuracy needs. However, we expect that by enabling AP GRF estimates in the community, new thresholds that indicate meaningful differences in estimation accuracy will emerge as more studies are conducted in ecologically relevant contexts.

### D. Multi-Modal Models Estimate Propulsion Changes During Active Gait Rehabilitation

Towards the ultimate goal of enabling gait rehabilitation in unconstrained environments for people post-stroke [13], multi-modal estimation outperformed both IMU only and Insole only estimation in a number of test cases. We found that multi-modal estimation performed best during active gait training with a resistive exosuit or an FES neuroprosthesis. In addition to monitoring biomechanical responses, wearable sensors have been used to inform parameters of robotic systems for healthy individuals [73], people post-stroke [5], and individuals with cerebral palsy [74]. Similarly, FES profiles for individuals with incomplete SCI have also been defined using IMU-based measures [75]. Thus, this work opens the possibility of leveraging accurate estimates of propulsion or other related biomechanical metrics to further inform rehabilitation parameters with active gait interventions.

### E. Limitations and Future Directions

Although these results are promising, there are several limitations to acknowledge to understand the scope of this work. We elected to develop individualized models to account for the high variability in this population, but this requires individual-level data from any new person. Our investigation into the use of healthy data to augment smaller datasets from individuals post-stroke aimed to account for the reduced dataset sizes from clinical participants, but further study is required to quantify the implications of data augmentation for setting minimum data collection requirements. Leveraging unsupervised learning techniques such as unsupervised domain adaptation and manifold embeddings may further reduce data requirements for model development in this population [76].

This work was also conducted through a series of single-session data collections, and the model’s performance across days was not evaluated. Sensor positions may significantly vary on different days, which could adversely affect model performance. Future work may involve collecting and including data with varying sensor placements, or randomly shifting and rotating the current training data as a form of data augmentation, to further improve model robustness [77]. For the intended purpose of tracking propulsion throughout the course of rehabilitation, additional study is required to understand the sensitivity of these algorithms to day-to-day variability in individuals. For example, model performance may decline as a person’s gait pattern changes over time during rehabilitation, and thus may require continuous or frequent updates to the model parameters. Future work may use data collected before and after longitudinal gait training to estimate AP GRF during the training process [10]. Incorporating physics-driven features or task-specific constraints into the model may also enhance the generalizability of the model without the need for additional training data [78], [79]. Moreover, leveraging open-source datasets that include a large number of individuals with diverse health conditions could be useful for creating generalized models [80], [81].

In this work, we used a high-resolution commercial pressure insole. However, insoles can vary in their underlying sensing mechanism, spatial resolution, mechanical properties, and accuracy. Thus, directly applying a model trained on a new insole sensor would likely result in reduced model performance. In addition to transfer learning, future work may investigate simulating new sensors either as a subset of high-resolution sensor data or from force plate data [82]. Another approach may be to apply a teacher-student network approach, where a teacher model from one sensor guides the training of a student model for a different sensor [83].

Finally, while we aimed to capture a wide range of impairment levels in our clinical cohort, all our participants were in the chronic phase of recovery. Motor recovery occurs much more rapidly during the acute phase of stroke [84], so future work should determine whether this method is still feasible in these populations.

## V. Conclusion

In conclusion, this work aims to develop a method for propulsion estimation that enables clinically viable monitoring of paretic limb mechanics. We show that leveraging data from two fundamentally different measurement sources, representing kinematic and kinetic information, enables improved estimation accuracy and generalizability of key propulsion metrics. We further demonstrate the feasibility of this approach for tracking changes in propulsion with different populations, speeds, environments, and gait training interventions. We expect this work will provide an analytical tool to support and enable future community-based rehabilitation programs for people post-stroke. Altogether, these findings support the benefit of combining IMUs and insoles for propulsion estimation in real-world applications in the context of post-stroke gait rehabilitation.

## Supplementary Material

tnsre-3577961-mm

This article has supplementary downloadable material available at https://doi.org/10.1109/TNSRE.2025.3577961, provided by the authors.

## Figures and Tables

**TABLE I:** Participant Information Across Datasets. ^1^ Wore an Ankle-Foot-Orthosis on the Paretic Limb During the Experiment. ^2^ Used a Cane During the Experiment. Note That Some Participants Are Repeated, but Are Listed Separately Due to Potential Changes in Anatomical Parameters

	Subject ID Dataset	Height (cm)	Weight (kg)	Age (yrs)	Sex	Paretic Side	CWS (m/s)	No. of Trials
**Clinical Subjects**	S1 (*D*_1*C*_)	188	81.3	58	M	L	0.8	4
	S2 (*D*_2*C*_)	169	110	60	M	L	0.55	3
	S3 (*D*_3*C*_)	181	100.5	37	M	R	1.00	6
	S4 (*D*_4*C*_)	175	84.5	50	M	L	0.9	4
	S5^1^ (*D*_5*C*_)	167	126	58	F	R	0.5	6
	S2 (*D*_6*C*_)	169	110	60	M	L	0.55	5
	S6^1,2^ (*D*_7*C*_)	178	87.4	59	M	R	0.5	3
**Healthy Subjects**	S7 (*D*_1*H*_)	167	58.1	29	F	N/A	N/A	5
	S8 (*D*_2*H*_)	164	61.69	27	F	N/A	N/A	5
	S9 (*D*_3*H*_)	182.9	79.3	36	M	N/A	N/A	5
	S10 (*D*_4*H*_)	183	84	29	M	N/A	N/A	5
	S11 (*D*_5*H*_)	169.5	61.3	24	F	N/A	N/A	3
	S12 (*D*_6*H*_)	194.2	109.9	24	M	N/A	N/A	5
	S13 (*D*_7*H*_)	170	49.5	24	F	N/A	N/A	5
**Speed Demo**	S3	185	96.6	39	M	R	0.95	7
	S6^1,2^	180	71.3	60	M	R	0.35	4
	S14	178	74.9	65	M	L	0.65	8
**Exo/FES Demo**	S5^2^	167	123.8	60	F	R	0.35	4
	S14	178	74.9	65	M	L	0.65	4
	S15^2^	174	62.6	67	M	L	0.40	4

**TABLE II:** Feasibility of Using Mostly Treadmill Data to Train Models for Tracking Paretic Propulsion During Unconstrained Walking. RMSE and R^2^ Values Are From the Test Set Comprising Overground Walking at a Self-Selected Comfortable Speed. S3 Is a High-Level Participant, S6 Is a Low-Level Participant, and S14 Is a Mid-Level Participant. No Outdoor Data Was Collected for S6, So We Only Present Results From S3 and S14 Out of the Lab

Subject ID	IMU Only		Insole Only		IMU + Insole	
	RMSE (%bw)	R^2^	RMSE (%bw)	R^2^	RMSE (%bw)	R^2^
S3 (High)	1.74	0.95	2.49	0.90	2.05	0.95
S6 (Low)	1.62	0.58	2.31	0.72	0.94	0.85
S14 (Mid)	2.72	0.85	3.24	0.81	2.87	0.86

		Average peak paretic propulsion across speeds (%bw) / Estimated walking speed (m/s)					
		In-lab (from force plates & motion capture)			Real-World Estimated		
		Slow	Med	Fast	Slow	Med	Fast
S3	Pk Prop	5.88	10.12	16.45	6.72	10.27	14.48
	Walking Speed	0.81	1.14	1.53	0.51	0.75	1.05
S14	Pk Prop	3.52	5.09	7.88	4.81	5.90	7.42
	Walking Speed	0.76	0.97	1.27	0.61	0.70	0.93

**TABLE III:** Feasibility of Tracking Propulsion During Gait Interventions Conducted in Unconstrained Walking. All Models Were Trained and Validated With Treadmill Data, and Tested During Overground Walking in the Lab. Baseline Results Include the Full Minute of Pre-Exposure While the Active Results Include the Full Two Minutes of Active Intervention

Subject ID	IMU Only		Insole Only		IMU + Insole	
	Baseline	Active	Baseline	Active	Baseline	Active
S5	N/A	1.48/0.90	N/A	2.12/0.88	N/A	1.30/0.92
S14	2.61 / 0.93	2.53 / 0.90	2.57 / 0.96	3.08 / 0.83	1.56 / 0.96	2.02 / 0.93
S15	1.80 / 0.90	1.36 / 0.94	2.57 / 0.74	2.41 / 0.70	1.48 / 0.94	1.06 / 0.97
