# Supplementary material for: Multi-Modal Sensing for Propulsion Estimation in People Post-Stroke Across Speeds
Source: IEEE Trans Neural Syst Rehabil Eng. Author manuscript; Available in PMC 2025 Jul 20. (PMC12276930; doi:10.1109/TNSRE.2025.3577961)
Supplement: tnsre-3577961-mm [file NIHMS2090147-supplement-tnsre-3577961-mm.zip › tnsre-3577961-mm/08062025110858_TNSRE3577961SIFinal.docx]

Multi-modal sensing for propulsion estimation in people post-stroke across speeds – Supplemental Information

**Authors:** Krithika Swaminathan^1^*, Dabin K. Choe^1^*, Daekyum Kim^1,2,3^*, Flore Barde^1^, Teresa C. Baker^4^, Nicholas C. Wendel^4^, Andrew Chin^1^, Gregoire Bergamo^1^, Christopher J. Siviy^1^, Christina Lee^1^, Louis N. Awad^4^, Terry D. Ellis^4^, Conor J. Walsh^1^**

**Affiliations:**

^1^John A. Paulson School of Engineering and Applied Sciences, Harvard University; Cambridge, Massachusetts, USA.

^2^School of Mechanical Engineering, Korea University; Seoul, South Korea.

^3^School of Smart Mobility, Korea University, Seoul, Republic of Korea.

^4^Department of Physical Therapy, Boston University; Boston, Massachusetts, USA.

**Keywords:** wearable sensing, estimation, machine learning, gait biomechanics

* These authors contributed equally to this work
** Corresponding author


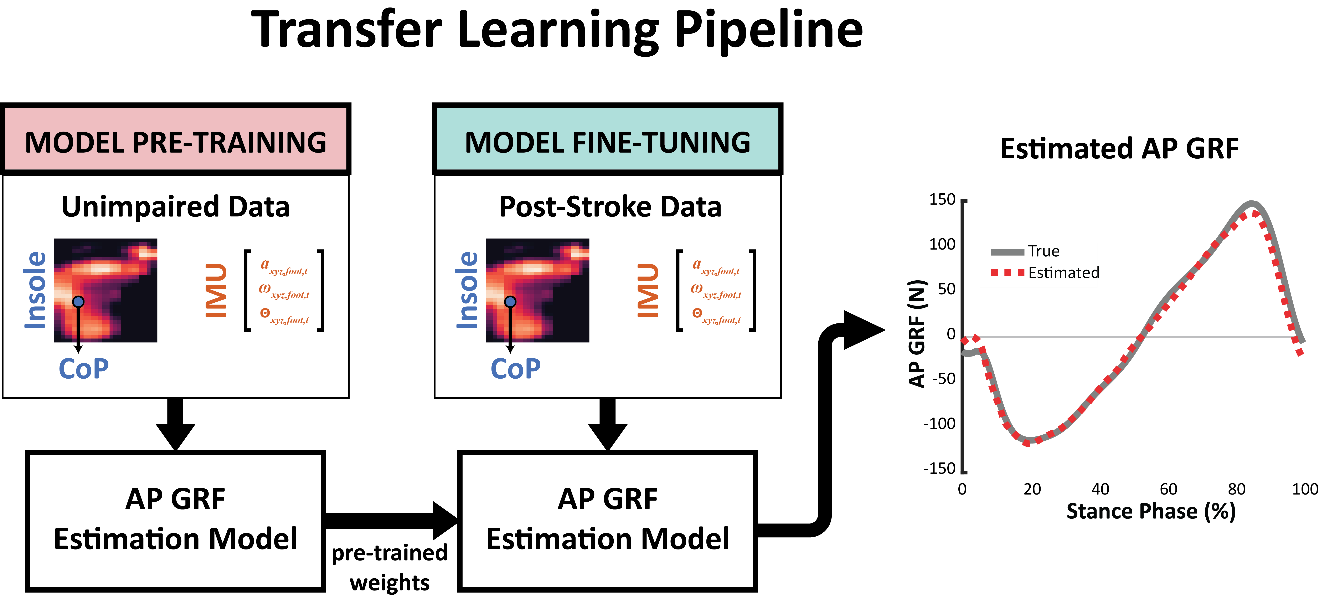


**Figure S1.** Transfer learning pipeline between healthy and clinical datasets. All IMU + Insole data from the healthy cohort was used to pre-train an estimation model, after which IMU + Insole data from each clinical participant was used to fine-tune the individualized model weights before generating the final AP GRF estimate.

|  | **Dataset** | **IMU Only** | | **Insole Only** | | **IMU + Insole** | |
| --- | --- | --- | --- | --- | --- | --- | --- |
|  |  | **RMSE/NRMSE** | **R2** | **RMSE/NRMSE** | **R2** | **RMSE/NRMSE** | **R2** |
| **Clinical Subjects** | S1 ($D_{1C}$) | 1.12/4.22 | 0.97 | 1.59/5.98 | 0.93 | **0.87/3.25** | **0.98** |
|  | S2 ($D_{2C}$) | 1.21/5.57 | 0.95 | 1.16/5.36 | 0.96 | **0.97/4.49** | **0.97** |
|  | S3 ($D_{3C}$) | 1.19/3.37 | 0.98 | 1.65/4.68 | 0.97 | **0.79/2.25** | **0.99** |
|  | S4 ($D_{4C}$) | 1.12/4.63 | 0.94 | 1.44/5.93 | 0.91 | **0.90/3.73** | **0.96** |
|  | S5^1^ ($D_{5C}$) | 0.96/4.35 | 0.96 | 0.88/4.01 | 0.97 | **0.70/3.17** | **0.98** |
|  | S2 ($D_{6C}$) | 1.27/5.99 | 0.94 | **0.85/4.00** | **0.98** | 0.89/4.21 | 0.97 |
|  | S6^1,2^ ($D_{7C}$) | 0.56/5.73 | 0.95 | 0.61/6.15 | 0.94 | **0.49/5.02** | **0.96** |
|  | **Mean**  **(Std Dev)** | 1.06/4.84  (0.24/0.96) | 0.96 (0.02) | 1.17/5.16  (0.40/0.93) | 0.95 (0.02) | **0.80/3.73**  **(0.16/0.93)** | **0.97**  **(0.01)** |
| **Healthy Subjects** | S7 ($D_{1H}$) | 1.11/2.22 | 0.99 | 1.62/3.26 | 0.98 | **0.91/1.83** | **0.99** |
|  | S8 ($D_{2H}$) | 0.94/2.20 | 0.99 | 1.83/4.27 | 0.96 | **0.80/1.88** | **0.99** |
|  | S9 ($D_{3H}$) | 1.09/2.66 | 0.99 | 1.77/4.35 | 0.98 | **0.77/1.88** | **0.99** |
|  | S10 ($D_{4H}$) | **0.86/2.04** | **0.99** | 2.08/4.92 | 0.98 | 0.93/2.21 | 0.99 |
|  | S11 ($D_{5H}$) | 1.28/3.69 | 0.98 | 4.90/14.16 | 0.79 | **0.62/1.78** | **0.99** |
|  | S12 ($D_{6H}$) | 1.07/2.20 | 0.99 | 2.14/4.38 | 0.96 | **0.86/1.76** | **0.99** |
|  | S13 ($D_{7H}$) | 1.25/2.84 | 0.98 | 2.90/6.56 | 0.93 | **1.10/2.49** | **0.99** |
|  | **Mean**  **(Std Dev)** | 1.09/2.55  (0.15/0.58) | 0.99 (0.01) | 2.46/5.99  (1.15/3.74) | 0.94  (0.07) | **0.86/1.98**  **(0.15/0.27)** | **0.99 (0.00)** |

**Table S1.** Performance of individual-specific models across the stance phase using different sensor combinations. Bolded values represent the best performance across the three sensor combinations for each individual and on average. Error metrics are computed for the paretic side in the clinical cohort and the left leg for the healthy cohort. ^1^Wore an ankle-foot-orthosis on the paretic limb during the experiment. ^2^Used a cane during the experiment.

|  | **Dataset** | **IMU Only** | | **Insole Only** | | **IMU + Insole** | |
| --- | --- | --- | --- | --- | --- | --- | --- |
|  |  | **RMSE/NRMSE** | **R2** | **RMSE/NRMSE** | **R2** | **RMSE/NRMSE** | **R2** |
| **Clinical Subjects** | S1 ($D_{1C}$) | 1.17/2.95 | 0.98 | 1.31/3.29 | 0.98 | **0.81/2.03** | **0.99** |
|  | S2 ($D_{2C}$) | 0.97/4.39 | 0.97 | 1.09/4.91 | 0.96 | **0.71/3.21** | **0.98** |
|  | S3 ($D_{3C}$) | 1.44/3.89 | 0.98 | **1.31/3.55** | **0.98** | 2.14/5.80 | 0.98 |
|  | S4 ($D_{4C}$) | 0.93/3.49 | 0.96 | 1.51/5.65 | 0.92 | **0.92/3.45** | **0.97** |
|  | S5^1^ ($D_{5C}$) | 0.90/3.76 | 0.97 | **0.69/2.89** | **0.98** | 0.72/3.03 | 0.98 |
|  | S2 ($D_{6C}$) | 0.81/3.48 | 0.98 | 0.91/3.94 | 0.97 | **0.65/2.82** | **0.99** |
|  | S6^1,2^ ($D_{7C}$) | 0.56/3.33 | 0.97 | 0.74/4.40 | 0.96 | **0.50/3.00** | **0.98** |
|  | **Mean**  **(Std Dev)** | 0.97/3.61  (0.28/0.46) | 0.97  (0.01) | 1.08/4.09  (0.31/0.97) | 0.96  (0.02) | **0.92/3.33 (0.55/1.18)** | **0.98**  **(0.01)** |
| **Healthy Subjects** | S7 ($D_{1H}$) | 1.07/2.21 | 0.99 | 2.63/5.42 | 0.96 | **0.78/1.61** | **0.99** |
|  | S8 ($D_{2H}$) | 1.04/2.51 | 0.99 | 2.13/5.15 | 0.96 | **0.92/2.22** | **0.99** |
|  | S9 ($D_{3H}$) | 1.07/2.58 | 0.99 | 1.29/3.11 | 0.98 | **1.00/2.40** | **0.99** |
|  | S10 ($D_{4H}$) | **1.47/3.05** | **0.98** | 2.25/4.69 | 0.97 | 1.64/3.42 | 0.99 |
|  | S11 ($D_{5H}$) | 1.51/4.21 | 0.97 | 2.63/7.32 | 0.94 | **0.78/2.18** | **0.99** |
|  | S12 ($D_{6H}$) | **0.96/2.07** | **0.99** | 2.69/5.80 | 0.94 | 0.97/2.10 | 0.99 |
|  | S13 ($D_{7H}$) | 1.08/2.60 | 0.98 | 4.65/11.22 | 0.89 | **0.87/2.09** | **0.99** |
|  | **Mean**  **(Std Dev)** | 1.17/2.75  (0.22/0.72) | 0.99  (0.01) | 2.61/6.10  (1.02/2.58) | 0.95  (0.03) | **0.99/2.29**  **(0.30/0.56)** | **0.99**  **(0.00)** |

**Table S2.** AP GRF estimation results for the contralateral limb. Bolded values represent the best performance across the three sensor combinations for each individual and on average. These results represent model performance on the non-paretic leg for the clinical cohort and the right leg for the healthy cohort. ^1^Wore an ankle-foot-orthosis on the paretic limb during the experiment. ^2^Used a cane during the experiment.


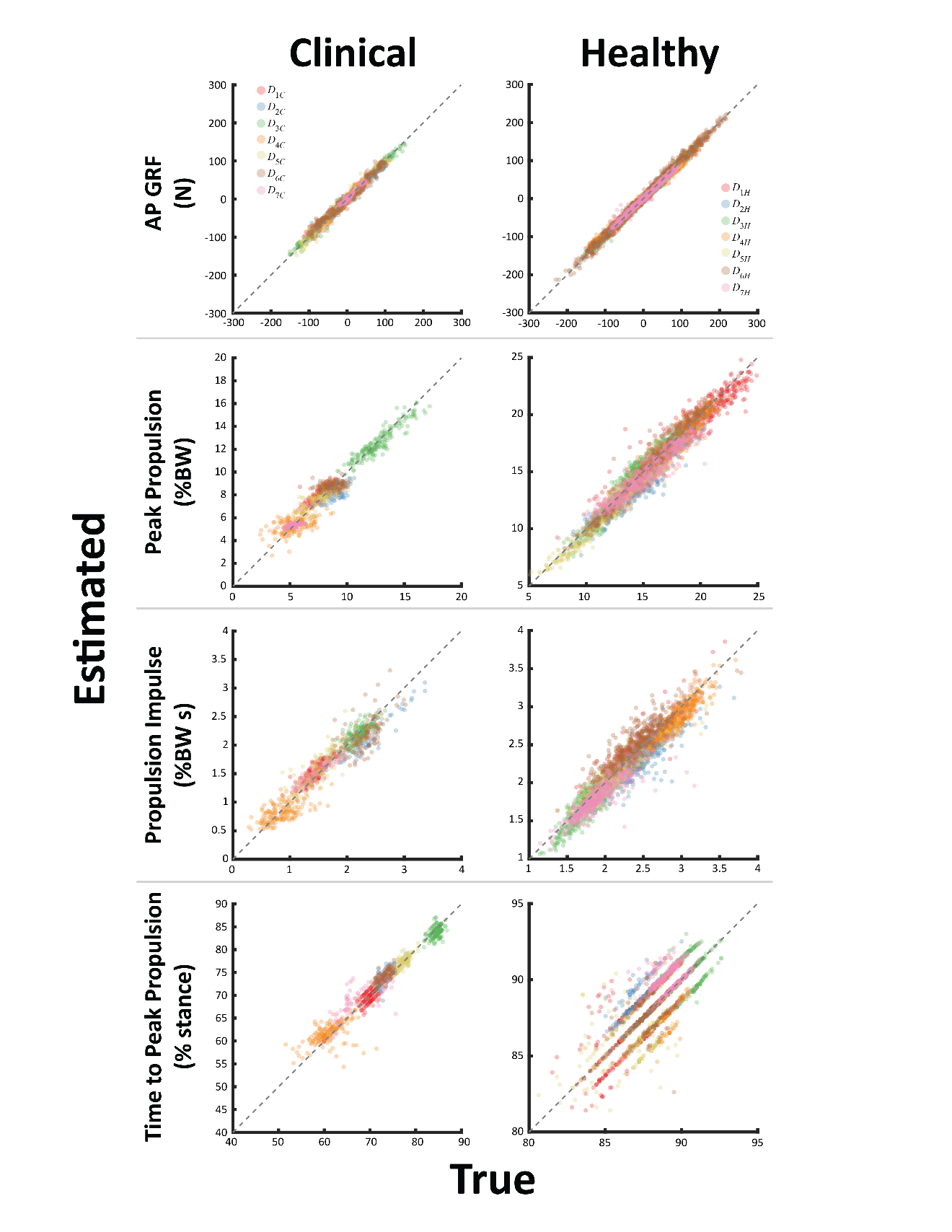


**Figure S2. IMU + Insole AP GRF Estimation Results.** Correlation plots for time series and key propulsion point metrics for each clinical and healthy dataset. For AP GRF time series data, we sample every 100^th^ point from the healthy test sets and every 50^th^ point from the clinical test sets to improve the interpretability of visualization. We note that the maximum resolution of peak propulsion timing is 0.1 %stance. Data represents performance on the paretic side for the clinical datasets and on the left side for the unimpaired datasets.

| **Dataset** | **Pk Prop (%BW)** | **Prop Imp (%BW s)** | **Pp**  **(%)** | **Pk Prop Time (%stance)** |
| --- | --- | --- | --- | --- |
| S1 ($D_{1C}$) | 0.56 | 0.13 | 1.79 | 1.07 |
| S2 ($D_{2C}$) | 1.01 | 0.28 | 2.40 | 1.61 |
| S3 ($D_{3C}$) | 0.61 | 0.12 | 8.40 | 1.26 |
| S4 ($D_{4C}$) | 0.97 | 0.21 | 5.05 | 2.81 |
| S5^1^ ($D_{5C}$) | 0.66 | 0.19 | 1.92 | 0.86 |
| S2 ($D_{6C}$) | 0.76 | 0.26 | 1.93 | 1.38 |
| S6^1,2^ ($D_{7C}$) | 0.40 | 0.13 | 3.02 | 3.31 |
| **Mean** | 0.71 (0.22) | 0.19 (0.07) | 3.50 (2.44) | 1.76 (0.93) |

**Table S3.** Model performance (RMSE) across individual-specific models for estimating point metrics with IMU + Insole input. ^1^Wore an ankle-foot-orthosis on the paretic limb during the experiment. ^2^Used a cane during the experiment.

| **Dataset** | **No Fine-Tuning** | | | **With Fine-Tuning** | | |
| --- | --- | --- | --- | --- | --- | --- |
|  | **RMSE/NRMSE** | **R2** | **Pk Prop** | **RMSE/NRMSE** | **R2** | **Pk Prop** |
| S1 ($D_{1C}$) | 0.91/3.41 | 0.98 | 0.53 | 0.92/3.46 | 0.98 | 0.56 |
| S2 ($D_{2C}$) | 0.91/4.19 | 0.98 | 0.81 | 1.10/5.07 | 0.96 | 1.15 |
| S3 ($D_{3C}$) | 1.15/3.26 | 0.98 | 1.18 | **0.94/2.67** | **0.99** | **0.89** |
| S4 ($D_{4C}$) | 1.14/4.71 | 0.94 | 1.17 | **1.10/4.53** | **0.94** | **1.12** |
| S5^1^ ($D_{5C}$) | 0.80/3.62 | 0.97 | 0.68 | **0.71/3.21** | **0.98** | **0.68** |
| S2 ($D_{6C}$) | 1.08/5.06 | 0.96 | 0.81 | **0.73/3.44** | **0.98** | **0.59** |
| S6^1,2^ ($D_{7C}$) | 0.54/5.45 | 0.96 | 0.50 | 0.54/5.46 | 0.95 | 0.70 |
| **Mean**  **(Std Dev)** | 0.93/4.24  (0.22/0.85) | 0.97  (0.02) | 0.81  (0.28) | **0.86/3.98**  **(0.21/1.04)** | 0.97  (0.02) | 0.82  (0.24) |

**Table S4.** Effects of transfer learning on AP GRF estimator performance with reduced datasets. IMU + Insole data were used as input to the models and the last set of weights from pre-training (Fig. S1) were used to initialize the individualized models. The last 50 percent of the training and validation datasets were used to fine-tune the models (“With Fine-Tuning”) or train the models from scratch (“No Fine-Tuning”). Bolded values indicate improvements relative to model performance without fine-tuning. ^1^Wore an ankle-foot-orthosis on the paretic limb during the experiment. ^2^Used a cane during the experiment.

| **Dataset** | **Best-Pretrained Weights** | | | **Last-Pretrained Weights** | | |
| --- | --- | --- | --- | --- | --- | --- |
|  | **RMSE/NRMSE** | **R2** | **Pk Prop** | **RMSE/NRMSE** | **R2** | **Pk Prop** |
| S1 ($D_{1C}$) | 0.94/3.53 | 0.98 | 0.77 | 0.87/3.26 | 0.98 | 0.58 |
| S2 ($D_{2C}$) | **0.89/4.11** | 0.97 | **0.95** | 1.00/4.62 | 0.97 | 1.09 |
| S3 ($D_{3C}$) | 0.92/2.60 | 0.99 | 0.81 | 0.89/2.54 | 0.99 | 0.91 |
| S4 ($D_{4C}$) | 1.00/4.11 | 0.95 | **0.95** | 1.14/4.72 | 0.94 | 1.15 |
| S5^1^ ($D_{5C}$) | 0.75/3.42 | 0.98 | 0.71 | **0.62/2.80** | 0.98 | **0.58** |
| S2 ($D_{6C}$) | **0.71/3.35** | 0.98 | **0.65** | **0.67/3.14** | 0.99 | **0.57** |
| S6^1,2^ ($D_{7C}$) | 0.54/5.52 | 0.96 | 0.79 | 0.53/5.41 | 0.95 | 0.93 |
| **Mean**  **(Std Dev)** | 0.82/3.80  (0.16/0.91) | 0.97  (0.01) | 0.80  (0.11) | 0.82/3.78  (0.22/1.11) | 0.97  (0.02) | 0.83  (0.25) |

**Table S5.** Effects of transfer learning on AP GRF estimator performance using IMU + Insole inputs with 500 epochs of fine-tuning using the best or last set of weights from pre-training (Fig. S1) for model initialization. The “Best-Pretrained Weights” are the best performing weights on the healthy validation data and the “Last-Pretrained Weights” represent the best performing weights on the healthy training data. Bolded values indicate improvements relative to model performance without transfer learning. ^1^Wore an ankle-foot-orthosis on the paretic limb during the experiment. ^2^Used a cane during the experiment.

| **Subject ID** | **Peak paretic propulsion during different training periods (%BW)** | | | | | |
| --- | --- | --- | --- | --- | --- | --- |
|  | **In-lab (TM - Validation)** | | **In-lab (OG - Test)** | | **Real-World Estimated** | |
|  | **Baseline** | **Active** | **Baseline** | **Active** | **Baseline** | **Active** |
| S5 | 6.00 | 5.29 | - | 5.50 | 3.27 | 5.30 |
| S14 | 4.92 | 7.18 | 4.91 | 6.04 | 6.43 | 7.62 |
| S15 | 4.93 | 5.39 | 5.68 | 5.53 | 4.09 | 3.95 |

**Table S6.** Feasibility of tracking propulsion during gait interventions conducted in unconstrained walking with an IMU + Insole model. In-lab data are obtained from ground truth force plates while real-world data are estimated. Baseline results include the full minute of pre-exposure while the Active results include the full two minutes of active intervention. In-lab data is left blank if there were no good strides of force plate data for analysis.
